# Transcutaneous Carbon Dioxide Induces Mitochondrial Apoptosis and Suppresses Metastasis of Oral Squamous Cell Carcinoma *In Vivo*

**DOI:** 10.1371/journal.pone.0100530

**Published:** 2014-07-02

**Authors:** Daisuke Takeda, Takumi Hasegawa, Takeshi Ueha, Yusuke Imai, Akiko Sakakibara, Masaya Minoda, Teruya Kawamoto, Tsutomu Minamikawa, Yasuyuki Shibuya, Toshihiro Akisue, Yoshitada Sakai, Masahiro Kurosaka, Takahide Komori

**Affiliations:** 1 Department of Oral and Maxillofacial Surgery, Kobe University Graduate School of Medicine, Kobe, Japan; 2 NeoChemir, Kobe, Japan; 3 Department of Orthopaedic Surgery, Kobe University Graduate School of Medicine, Kobe, Japan; 4 Division of Rehabilitation Medicine, Kobe University Graduate School of Medicine, Kobe, Japan; Broad Institute of Harvard and MIT, United States of America

## Abstract

Squamous cell carcinoma (SCC) is the main histological type of oral cancer. Its growth rate and incidence of metastasis to regional lymph nodes is influenced by various factors, including hypoxic conditions. We have previously reported that transcutaneous CO_2_ induces mitochondrial apoptosis and decreases lung metastasis by reoxygenating sarcoma cells. However, previous studies have not determined the sequential mechanism by which transcutaneous CO_2_ suppresses growth of epithelial tumors, including SCCs. Moreover, there is no report that transcutaneous CO_2_ suppresses lymphogenous metastasis using human cell lines xenografts. In this study, we examined the effects of transcutaneous CO_2_ on cancer apoptosis and lymphogenous metastasis using human SCC xenografts. Our results showed that transcutaneous CO_2_ affects expressions of PGC-1α and TFAM and protein levels of cleavage products of caspase-3, caspase-9 and PARP, which relatives mitochondrial apoptosis. They also showed that transcutaneous CO_2_ significantly inhibits SCC tumor growth and affects expressions of HIF-1α, VEGF, MMP-2 and MMP-9, which play essential roles in tumor angiogenesis, invasion and metastasis. In conclusion, transcutaneous CO_2_ suppressed tumor growth, increased mitochondrial apoptosis and decreased the number of lymph node metastasis in human SCC by decreasing intra-tumoral hypoxia and suppressing metastatic potential with no observable effect *in vivo*. Our findings indicate that transcutaneous CO_2_ could be a novel therapeutic tool for treating human SCC.

## Introduction

Squamous cell carcinoma (SCC), the main histological type of oral cancer, comprises over 90% of all oral malignancies. SCCs grow rapidly and readily metastasize to the regional lymph nodes [1]. Lymph node metastasis is a well-established negative prognostic indicator in the treatment of squamous cell carcinoma of the head and neck [2]. Considerable efforts have been made to control both metastases and primary tumors in patients with oral cancer [3].

Hypoxia, a common feature of malignant tumors, occurs in numerous solid tumors [4]. Hypoxia inducible factors (HIFs) depends directly on tumor hypoxia and, in human malignancies, HIFs critically affect resistance to chemo- and radiation therapy and are associated with histological grade, stage, and recurrence [5]. Overexpression of HIF-1α, an oxygen-dependent α subunit of HIF, has been associated with tumor cell growth, lymph node metastasis and survival in head and neck tumors [6]–[8]. HIF-1α mediates the adaptation of cancer cells to hypoxic environments by controlling expression of hundreds of genes, including vascular endothelial growth factor (VEGF). HIF-1α is also strongly induced under hypoxic conditions, and activates transcription of genes involved in metastasis, including matrix metalloproteinases (MMPs). Increased expression of certain MMPs correlates with tumor growth, invasion, and poor prognosis in patients with malignancies. Among the MMP subtypes, MMP-2 and MMP-9 are widely reported as crucial to the invasive and metastatic potentials of various malignancies. Although decreasing activity of HIF-1α and/or MMPs by reducing tumor hypoxia could be an effective strategy in cancer treatment, there is currently no effective way of achieving reduction of hypoxia in tumors [5], [9].

The benefits of carbonated spa (carbon dioxide; CO_2_ therapy) have long been known in Europe. CO_2_ therapy is believed to an effective treatment for cardiac diseases and skin problems [10], [11]. The therapeutic effects of CO_2_ are mediated by an increase in blood flow and microcirculation, nitric oxide-dependent neocapillary formation, and an increase of partial pressure of O_2_ in the local tissue known as the Bohr effect. The Bohr effect is represented by a rightward shift of the O_2_–hemoglobin dissociation curve with increasing pCO_2_ or decreasing pH [12]. We have previously demonstrated *in vivo* that transcutaneous CO_2_ leads to increased O_2_ release from red blood cells, causing an “Artificial Bohr Effect” [12], and up-regulates O_2_ pressure, with increased expression of peroxisome proliferator-activated receptor gamma coactivator-1 alpha (PGC-1α) and the number of mitochondria in treated skeletal muscle tissue [13]. We have also shown that, in human malignant fibrous histiocytoma (MFH) xenografts, transcutaneous CO_2_ decreases expression of HIF-1α and induces mitochondrial apoptosis, in turn increasing PGC-1α and mitochondrial transcription factor A (TFAM) [9], [14]. In osteosarcoma, transcutaneous CO_2_ also decreases the incidence of hematogenous metastases to the lungs by reducing hypoxia [5].

Previous studies have not determined the sequential mechanism by which transcutaneous CO_2_ suppresses growth of epithelial tumors, including SCCs. Moreover, there is no report that transcutaneous CO_2_ suppresses lymphogenous metastasis using human cancer cell xenografts. MFH and osteosarcoma mainly metastasize hematogenously, however both hematogenous and lymphogenous metastases occur from SCCs. Lymphogenous metastasis, rather than hematogenous metastasis, is often observed in SCCs in clinical settings. Here, we suggest that transcutaneous CO_2_ may reduce hypoxia and induce mitochondrial pathways in SCC. In this study, we investigated whether transcutaneous CO_2_ can induce tumor cell apoptosis and suppress lymphogenous metastatic spread to the regional lymph nodes in human SCC.

## Materials and Methods

### Cell Culture

The oral cancer cell line used in this study, HSC-3, was obtained from the Health Science Research Resources Bank (Osaka, Japan). HSC-3 cells were established from a metastatic deposit of poorly differentiated SCC of the tongue in a mid-internal jugular lymph node from a 64 year old man [15]. HSC-3 cells in Eagle's minimum essential medium (Sigma-Aldrich, St Louis, MO, USA) supplemented with 10% fetal bovine serum (Sigma-Aldrich) and 1000 units/mL penicillin/streptomycin solution (Sigma-Aldrich) were routinely cultured in an incubator in 5% CO_2_ at 37°C. Trypsin (0.25%) and ethylenediaminetetraacetic acid (0.02%; Sigma-Aldrich) solution was used to isolate cells for subculture, as previously described [16].

### Animal models

Male athymic BALB/cAJcl–nu/nu nude mice aged 7 weeks were obtained from CLEA Japan (Tokyo, Japan). The animals were maintained under pathogen-free conditions, in accordance with institutional principles. All animal experiments were performed in accordance with the Guidelines for Animal Experimentation at Kobe University Animal Experimentation Regulations (Permission number: P120602) and were approved by the Institutional Animal Care and Use Committee. HSC-3 cells (2.0×10^6^ cells in 500 µL phosphate buffered saline [PBS]) were injected subcutaneously into the backs of the mice, as previously described [17].

### Transcutaneous CO_2_ treatment

**Figure 1:**
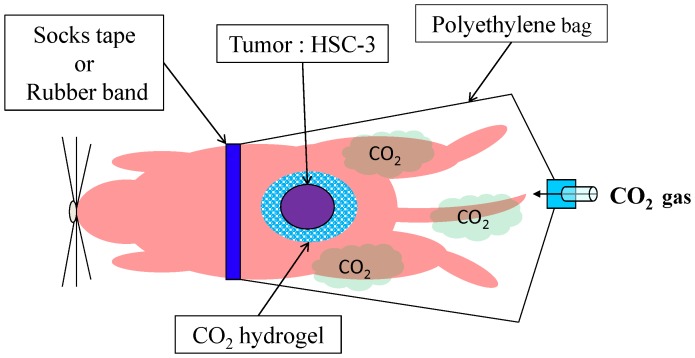
Procedure for administering transcutaneous CO_2_ in animal model of human SCC. The area of skin around the implanted tumor was covered with CO_2_ hydrogel and sealed with a polyethylene bag, through which 100% CO_2_ gas was administered. Treatment commenced 7 days after HSC-3 cell implantation, and was performed twice a week for 3 weeks.

Transcutaneous CO_2_ was administered as previously described. Briefly, the area of skin around the implanted tumor was covered with CO_2_ absorption-enhancing hydrogel (CO_2_ hydrogel). This area was then sealed with a polyethylene bag and 100% CO_2_ gas pumped into the bag (Fig. 1). Each treatment was performed for 20 min. Control animals were treated similarly, room air replacing the CO_2_ [5], [9], [13], [14].

### *In vivo* HSC-3 tumor studies

Sixteen mice were randomly divided into two groups: a CO_2_-treated group (*n* = 8) and a control group (*n* = 8). Treatment commenced 7 days after HSC-3 cell implantation and was performed twice a week for 3 weeks. Tumor volume and body weight were monitored twice weekly until the end of the treatment. Tumor volume was calculated as previously described according to the formula V = π/6×a^2^×b, where a and b represent the shorter and longer diameters of the tumor, respectively [5], [9], [14].

### Surgical procedure

The treatment was completed 4 weeks after commencing treatment, and 24 h after the end of treatment, mice were weighed and sacrificed by intraperitoneal injection of an overdose pentobarbital (Kyoritsuseiyaku, Tokyo, Japan) and tumors and bilateral axillary lymph nodes were removed. Immediately after dissection, single cell suspensions were processed from half the tumor, and RNA and protein were extracted. The other half of the tumor was formalin-fixed and paraffin-embedded for staining. Serial 10 µm thick transverse sections were prepared from each block.

### Apoptosis analysis: determination of the relative mitochondrial copy number

Genomic DNA was isolated from 10 µg of tumor using a GenElute Mammalian Genomic DNA Miniprep kit (Sigma-Aldrich). Mitochondrial DNA (mtDNA) content relative to PGC-1α and TFAM gene was measured using real-time polymerase chain reaction (PCR). The sequences of primer pairs specifically designed to amplify the entire D-loop of mtDNA were as follows: forward (5′-CTC CAC CAT TAG CAC CCA AAG-3′) and reverse (5′-GTG ATG TGA GCC CGT CTA AAC-3′) (D-loop, 1232 bp) and for nucleotide DNA-encoded internal control gene β-actin: forward (5′-ATC ATG TTT GAG ACC TTC AAC A-3′) and reverse (5′-CAT CTC TTG CTC GAA GTC CA-3′)(β-actin, 318 bp) [18]. The PCR cycling conditions were 15 min at 95°C, followed by 40 cycles of 30 sec at 95°C, 20 sec at 58°C and 2 min at 72°C.

### Analysis of mRNA expression of apoptotic and metastatic factors: total RNA extraction and reverse transcription

Total RNA was extracted from tumors using an RNeasy Mini Kit (Qiagen, Valencia, CA, USA). cDNA was synthesized (300 ng total RNA) using a High Capacity cDNA Transcription Kit (Applied Biosystems, Foster City, CA, USA).

### Quantitative real-time PCR

The mRNA expression of β-actin, PGC-1α, TFAM, VEGF, MMP-2 and MMP-9 were analyzed by quantitative real-time PCR. Quantification of mRNA transcription was performed using an Applied Biosystems StepOne Real-Time PCR System (Applied Biosystems). Real-Time PCR reactions (20 µL) contained 0.5 µM forward primer, 0.5 µM reverse primer, and 1 µL of cDNA template from RT reaction, and 10 µL (2×) master mix for Power SYBR green master mix (Applied Biosystems). Reaction conditions included 10 min at 95°C, followed by 40 cycles of 15 sec at 95°C and 1 min at 60°C. The level of each target gene was normalized to β-actin level and expressed relative to the levels of the control group (ΔΔCT methods; Applied Biosystems).

### Design of primers

Primers for β-actin, the housekeeping gene, were designed as follows: forward (5′-GAT GAG ATT GGC ATG GCT TT -3′) and reverse (5′-CAC CTT CAC CGT TCC AGT TT -3′), for PGC-1α: forward (5′-GGC AGA AGG CAA TTG AAG AG -3′) and reverse (5′-TCA AAA CGG TCC CTC AGT TC -3′), for TFAM: forward (5′-GTG TAT TGC CAG GAG GCT CT -3′) and reverse (5′-CAC ATG CTT CGG AGA AAC G-3′), for VEGF: forward (5′-GAG CCT TGC CTT GCT GCT CTA C-3′) and reverse (5′-CAC CAG GGT CTC GAT TGG ATG -3′), for MMP-2: forward (5′-ACA GCA GGT CTC AGC CTC AT-3′) and reverse (5′-TGC CTC TGG ACA ACA CAG AC -3′) and for MMP-9: forward (5′-TCT TCC CTG GAG ACC TGA GA-3′) and reverse (5′-ATT TCG ACT CTC CAC GCA TC-3′). All primers were purchased from Invitrogen (Carlsbad, CA, USA).

### Western immunoblot analysis

Cell lysates were prepared from implanted tumor tissues using a whole cell lysis buffer supplemented with Halt protease and phosphatase inhibitor cocktail (Mammalian Protein Extraction Reagent; Thermo Scientific, Rockford, IL, USA). Protein samples were processed using standard western immunoblotting procedures. Membranes were incubated overnight at 4°C with the following antibodies in Can Get Signal Immunoreaction Enhancer Solution 1 (Toyobo, Osaka, Japan): anti-human cleaved caspase-3 antibody (1∶500) (Cell Signaling Technology, Danvers, MA, USA), anti-human cleaved caspase-9 antibody (1∶500) (Cell Signaling Technology), anti-human poly-ADP-ribose polymerase-1 (PARP) antibody (1∶500) (Cell Signaling Technology), and anti-human α-tubulin antibody (1∶5000) (Sigma-Aldrich). After washing, the membranes were incubated with the appropriate secondary antibody conjugated to horseradish peroxidase (GE Healthcare Bio-Sciences, Piscataway, NJ, USA) in Can Get Signal Immunoreaction Enhancer Solution 2, and exposed with ECL Prime Plus western blotting detection system reagent (GE Healthcare Bio-Sciences). The signals were detected using a Chemilumino analyzer LAS-3000 mini (Fujifilm, Tokyo, Japan) [9].

### Fluorescence activated cell scanning (FACS) assay

DNA fragmentation was evaluated using an APO-DIRECT Kit according to the manufacturer's protocol (BD Pharmingen, Franklin Lakes, NJ, USA). Briefly, the implanted tumors were excised, minced and filtered through a cell strainer (BD Falcon, Bedford, MA, USA) to obtain single cell suspensions. Erythrocytes were lysed in BD Pharm Lyse Lysing Buffer (BD Pharmingen) and the remaining cells pelleted and resuspended in PBS. Single cell suspensions were fixed with 1% (v/v) paraformaldehyde and resuspended in 70% (v/v) ice cold ethanol at a concentration of 1×10^6^ cells/mL. Each cell pellet was resuspended in 51.0 µL of DNA Labeling Solution (Reaction Buffer, 10.0 µL; terminal deoxynucleotidyl transferase enzyme, 0.75 µL; fluorescein isothiocyanate (FITC) deoxyuridine triphosphate (dUTP), 8.0 µL; distilled H_2_O, 32.25 µL) and incubated for 60 min at 37°C. FITC dUTP-labeled cells were analyzed by flow cytometry with a 488 nm argon laser (FACS Calibur, BD Pharmingen) [14].

### Immunohistochemical staining

Formalin-fixed and paraffin-embedded tumor sections were pretreated with citrate buffer for 40 min at 95°C, quenched with 0.05% H_2_O_2_, and incubated overnight at 4°C with the following primary antibodies in Can Get Signal Immunostain Solution A (Toyobo): rabbit anti-human VEGF polyclonal antibody (1∶50) (Abcam, Cambridge, UK), rabbit anti-human HIF-1α antibody (1∶50) (Cell Signaling Technology), mouse anti-human MMP-2 antibody (1∶100) (Novocastra Laboratories, New-castle, UK) and mouse anti-human MMP-9 antibody (1∶100) (Novocastra Laboratories). Following this treatment, sections were incubated with horseradish peroxidase-conjugated goat anti-mouse IgG polyclonal antibody (Nichirei Bioscience, Tokyo, Japan) for 30 min at room temperature. Signals were developed as a brown reaction product using peroxidase substrate 3′,3′-diaminobenzidine (Nichirei Bioscience). The sections were counterstained with hematoxylin and examined with a BZ-8000 confocal microscope (Keyence, Osaka, Japan) [5].

### Detection of micro metastases in the axillary lymph nodes

We evaluated the existence of lymphogenous metastasis per mouse, all of axillary lymph nodes were dissected. The formalin-fixed and paraffin-embedded lymph node sections were stained with hematoxylin and eosin.

### Detection of metastases by PCR analysis for the human β-globin gene

Genomic DNA was extracted from axillary lymph nodes using SepaGene (EIDIA, Tokyo, Japan) according to the protocol recommended by the manufacturer. Two sets of primers for the human β-globin gene were designed (human β-globin Primer Set; Takara Bio, Shiga, Japan). The first primers were designed outside the region amplified by the second primer. The outer primers were designed as follows: GH20 (forward) (5′-GAA GAG CCA AGG ACA GGT AC-3′) and GH21 (reverse) (5′-GGA AAA TAG ACC AAT AGG CAG-3′) (amplified size of DNA: 408 bp); and the inner primers, KM29 (forward) (5′-GGT TGG CCA ATC TAC TCC CAG G-3′) and KM38 (reverse) (5′-TGG TCT CCT TAA ACC TGT CTT G-3′) (262 bp). The PCR reaction contents were as previously described using TaKaRa Taq (Takara Bio). The PCR conditions for the first reaction were 30 cycles of denaturing at 94°C for 1 min, annealing at 55°C for 2 min, and extension at 72°C for 2 min, according to the protocol recommended by the manufacturer. In the second step, the first PCR product was removed and reamplified using two sets of overlapping inner primers. Conditions for the second PCR were the same as for the first reaction. The PCR product was electrophoresed in a 2.0% agarose gel containing 0.5 µg/mL ethidium bromide [19]–[22].

### Statistical analysis

Data are presented as the mean values ± standard error. The results of the two groups were analyzed using the Mann–Whitney U-test. The level of statistical significance was set at P<0.05.

## Results

### Transcutaneous application of CO_2_ suppresses SCC growth by inducing apoptosis

**Figure 2:**
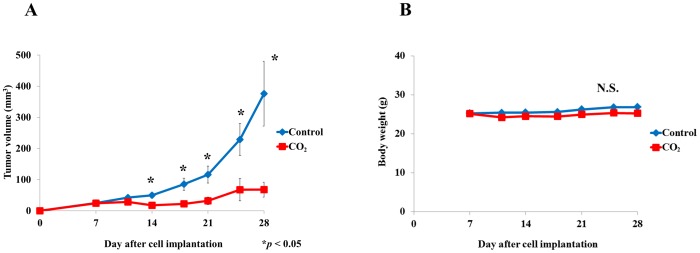
The effect of transcutaneous CO_2_ on HSC-3 tumor growth. Tumor growth (A) and body weight (B) was monitored for 4 weeks in the CO_2_-treated and control groups (*P<0.05).

**Figure 3:**
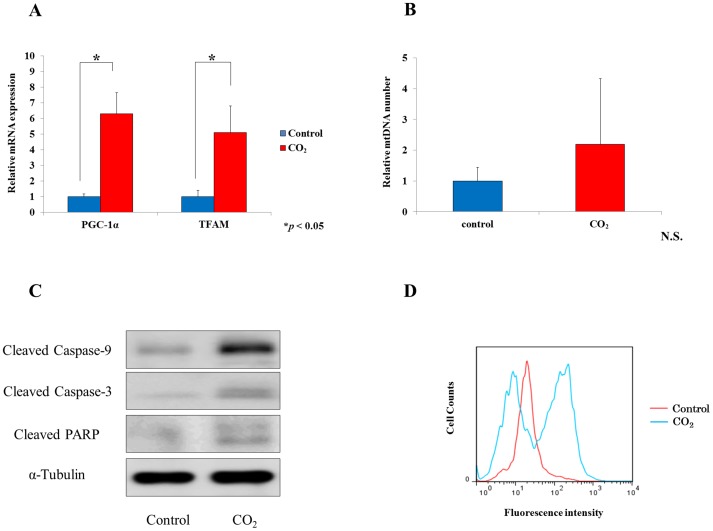
The effect of transcutaneous CO_2_ on HSC-3 cell apoptotic pathway. (A) The mRNA expressions of PGC-1α and TFAM gene were evaluated using real-time PCR (*P<0.05). (B) Mitochondrial DNA (mtDNA) content was also measured; differences were not statically significant. (C) Immunoblot analysis of expressions of the cleavage products of caspase-3, caspase-9 and PARP. β-actin was used as an endogenous loading control. (D) At the end of experiment, the numbers of apoptotic cells in tumors of both groups were assessed by FACS.

We evaluated the effect of transcutaneous CO_2_ on SCC cell growth. In the CO_2_-treated group, we found a significant decrease in tumor volume after 14 days (P<0.05, Fig. 2A). At the end of the experiment, tumor volume had decreased by significantly more in the CO_2_-treated (67.9±24.1 mm^3^) than in the control group (376.1±104.1 mm^3^, P<0.05, Fig. 2A). Mean body weight was slightly less in the CO_2_-treated than in the control group; however, this difference was not significant (Fig. 2B). Quantitative real-time PCR showed that mRNA expressions of PGC-1α and TFAM were significantly higher in the CO_2_-treated than in the control group (P<0.05, Fig. 3A). Mitochondrial copy number tended to be greater in the CO_2_-treated group; however, this difference was not significant (Fig. 3B). Immunoblot analysis showed greater amounts of cleavage products of caspase-3, caspase-9 and PARP in the CO_2_-treated than in the control group (Fig. 3C). FACS analysis showed that apoptotic cells were more numerous in the CO_2_-treated than in the control group (Fig. 3D).

### Transcutaneous CO_2_ significantly reduces intra-tumoral hypoxia and suppresses the potential to metastasize to lymph nodes

**Figure 4:**
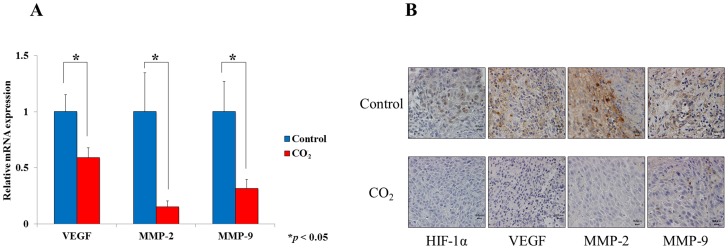
The effect of transcutaneous CO_2_ on the metastatic potential of HSC-3. (A) The mRNA expressions of VEGF, MMP-2 and MMP-9 gene were measured using real-time PCR (*P<0.05). (B) Immunohistochemical staining for HIF-1α, VEGF, MMP-2 and MMP-9 in implanted tumors from both groups.

Finally, we evaluated the effects of transcutaneous CO_2_ on tumor hypoxia and development of lymph nodes metastases in SCC. In implanted tumors, quantitative real-time PCR showed that mRNA expressions of VEGF, MMP-2 and MMP-9 were significantly lower in the CO_2_-treated than in the control group (P<0.05, Fig. 4A). HIF-1α, VEGF, MMP-2 and MMP-9 were barely detectable by immunohistochemical positive staining in the CO_2_-treated tumors, but were strongly positive in the control group (Fig. 4B).

### Transcutaneous CO_2_ significantly suppresses lymph node metastasis

**Figure 5:**
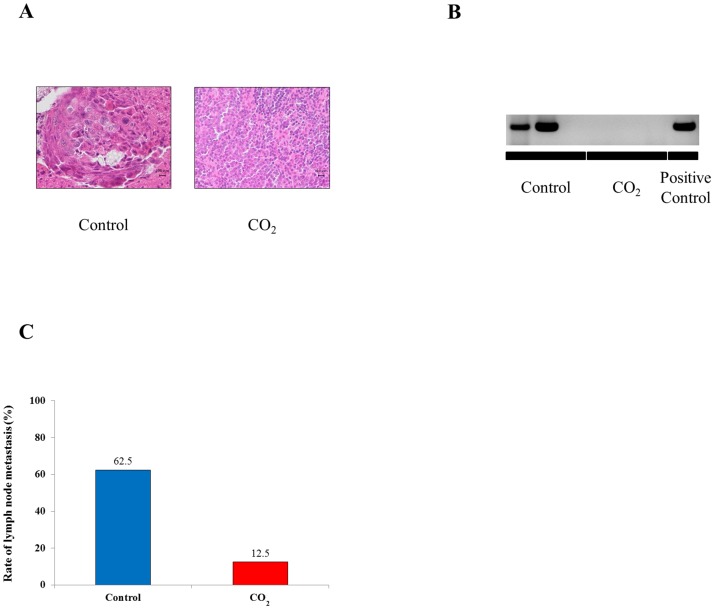
The effect of transcutaneous CO_2_ on metastasis of HSC-3 to the regional lymph nodes. (A) Detection of micrometastases in the axillary lymph nodes by hematoxylin and eosin staining. (B, C) Human β-globin gene expression in the axillary lymph nodes was assessed by PCR analysis to detect metastasis.

We identified micrometastatic lesions, so called cancroid pearls, in the control group's axillary lymph nodes, but not in those of the CO_2_-treated group (Fig. 5A). On PCR analysis, positive reactions with the human β-globin gene were observed in 5 of 8 mice in the control group (62.5%), but in only 1 of 8 mice in the CO_2_-treated group (12.5%) (Fig. 5B, C).

## Discussion

The prognosis of patients with advanced SCC remains poor because it is often hard to control lymph node metastasis [23]. Therefore, to improve prognosis, it is important not only to suppress tumor growth at the primary site but also to prevent lymph node metastasis. Surgical excision is the most effective therapy for SCC patients; however, resection of advanced disease can have serious cosmetic and functional consequences, making it difficult for these patients to be rehabilitated in a social sense. On the other hand, although chemotherapy and radiotherapy may cause less dysfunction, these therapies are often ineffective and associated with a poor prognosis because of local recurrence and metastasis [24]. Furthermore, when oral cancer has metastasized to lung, bone, or other distant organs, it is difficult to both achieve radical cure and maintain quality of life [16]. Although various combinations of surgery, chemo-radiotherapy and other treatments have been studied, there are numerous problems regarding effectiveness and adverse effects: further research is needed.

Hypoxia is common in solid tumors, including SCCs, and can increase the invasiveness and metastatic ability of tumor cells [25]. A hypoxic microenvironment induces various molecular pathways that allow tumor cells to become resistant to chemotherapy and radiotherapy. For example, expression of the multidrug resistance (MDR1) gene is dependent on hypoxia. This gene produces a protein that is associated with tumor resistance to chemotherapeutic agents [26]. In human oral SCC, a hypoxic microenvironment plays an important role in expressions of HIF-1α and MMPs and in proliferative activity of tumor cells [27]. The transcriptional factor HIF-1α, one of the key regulators of oxygen homeostasis, is an important mediator of solid tumor development *in vivo* because it promotes angiogenesis and inhibits cancer apoptosis. Overexpression of HIF-1α inhibits reduction of mitochondrial membrane potential by caspase-9 and caspase-3 [28]. HIF-1α also regulates VEGF expression, which has been shown to be a key mechanism in the abnormal and excessive angiogenesis and metastasis induced by hypoxia in malignant cells [9], [29], [30]. In hypoxic culture conditions, human SCC cell lines increase production of VEGF [31]. Because tumor angiogenesis plays an important role in local growth, enhancing cell motility, promoting metastasis and inhibition of apoptosis in oral cancer, inhibiting angiogenesis is an effective means in treating oral cancer [16]. The expression of VEGF has been shown to be strongly correlated with lymphangiogenesis and lymph node metastasis in oral SCC [32]. Additionally, the degree of VEGF expression has been reported as the most significant predictor of poor prognosis in oral and oropharyngeal SCCs [33]. HIF-1α also regulates MMPs that degrade extracellular matrix components and promote invasion [34]. Additionally, overexpression of MMPs has been shown to promote invasion and metastasis of various tumors, including oral cancer [35]–[38]. In oral SCC, the degree of activation of MMP-2 and MMP-9 is significantly higher in malignant tissues of patients with lymph node metastasis than in those without lymph node metastasis [39]. Therefore, improvement in hypoxic conditions may control tumor progression and decrease metastatic potential via regulating HIF-1α, VEGF and MMPs in SCC. Several therapeutic strategies, for example hyperbaric oxygen therapy, for changing oxygen conditions or specifically targeting hypoxic cells have been attempted. Unfortunately, these therapies have not been confirmed to be clinically beneficial.

Mitochondria play important roles in cellular energy metabolism and apoptosis. Alterations in respiratory activity and mtDNA mutations have been reported in various malignant tumors [40], [41]. Cancer cells produce their energy through the glycolytic pathway under anaerobic conditions [40]. We targeted the differences of mitochondrial energy metabolism between normal and cancer cells, and hypothesized that improving hypoxic and aerobic conditions should be useful and important for cancer treatment. Although mitochondria proliferate independently from cancer cells [42], the speed of cancer cell division and proliferation is more likely faster than mitochondria under hypoxic conditions. Therefore, while cancer cells exhibit abnormal proliferation,, the number of mitochondria decreases in cancer cells under hypoxic conditions, and mitochondrial dysfunction (the Warburg effect) results in avoiding apoptosis in tumor tissue. Apoptosis, a genetically encoded program for cell death that can be activated under physiological conditions, may be an important safeguard against tumor development [43]. Therefore, a current focus in cancer therapy research is improving understanding of apoptotic pathways and development of agents that target them [44]. We have also reported that an increased number of mitochondria induces apoptosis in sarcoma cells [45], and we speculate this phenomenon does not occur in normal tissue without cancer cell proliferation. In this study, we examined that transcutaneous CO_2_ inhibits SCC tumor growth *in vivo* by increasing the number of mitochondria and activating mitochondrial apoptosis by reducing intra-tumoral hypoxia.

We have previously shown that transcutaneous CO_2_ system causes absorption of CO_2_ and increase in O_2_ pressure in treated tissue, potentially causing an “Artificial Bohr Effect” [12]. CO_2_ therapy significantly decreases expressions of both HIF-1α and VEGF in human MFH, which suggests that CO_2_ therapy reduces hypoxia in the treated tumor tissue [9]. Based on these findings, we investigated whether making the environment of SCCs less hypoxic by using transcutaneous CO_2_ induces tumor cell apoptosis and suppresses lymphogenous metastasis to the regional lymph nodes by a mitochondrial pathway. In this study, we found that transcutaneous CO_2_ suppresses SCC growth by inducing apoptosis and significantly suppresses metastasis to lymph nodes by reducing intra-tumoral hypoxia and suppressing metastatic potential. These findings are consistent with our previous reports that transcutaneous CO_2_ alone significantly decreases tumor growth by inducing mitochondrial apoptosis in human MFH xenografts [14], and that transcutaneous CO_2_ decreases the incidence of lung metastasis of osteosarcoma by reducing local hypoxia [5]. Our findings strongly indicate that the mechanisms by which transcutaneous CO_2_ induces antitumor effects include suppressing metastasis of various malignant tumors and inducing tumor cell apoptosis, both mechanisms involving reduction of local hypoxia. However, whether decreasing the rate of metastasis is mediated by suppressing growth of the primary tumor or its ability to metastasize is not clear. Our future studies will focus on the investigation of lymphogenous metastatic factors.

It can be also considered that decreasing tumor growth results in reduced cancer cell viability and decreased micro-metastasis. We believe other mechanisms may mediate hypoxia-induced metastasis. Therefore, examining mobility or invasion in detail should be the focus of future studies.

## Conclusions

In summary, this is the first report to show that transcutaneous CO_2_ suppresses growth of primary human SCC and their lymphogenous metastasis to the regional lymph nodes by making their environment less hypoxic and increasing mitochondrial apoptosis. Our present findings indicate that transcutaneous CO_2_ decreases the malignant potential of SCC with few side effects.

## Supporting Information

Figure S1 **The effect of transcutaneous CO_2_ on the metastatic potential of HSC-3.** In an enlarged image of immunohistochemical staining, we observed that hypoxic condition (HIF-1α), abnormal excessive vascularization (VEGF) and metastatic potential (VEGF, MMP-2 and MMP-9) were decreased in CO_2_-treated cancer cells.
(TIF)
