# Supplementary figures and images for: Transcutaneous Carbon Dioxide Induces Mitochondrial Apoptosis and Suppresses Metastasis of Oral Squamous Cell Carcinoma In Vivo
Source: PLoS One. 2014 Jul 2;9(7):e100530. doi: 10.1371/journal.pone.0100530 (PMC4079455; doi:10.1371/journal.pone.0100530)

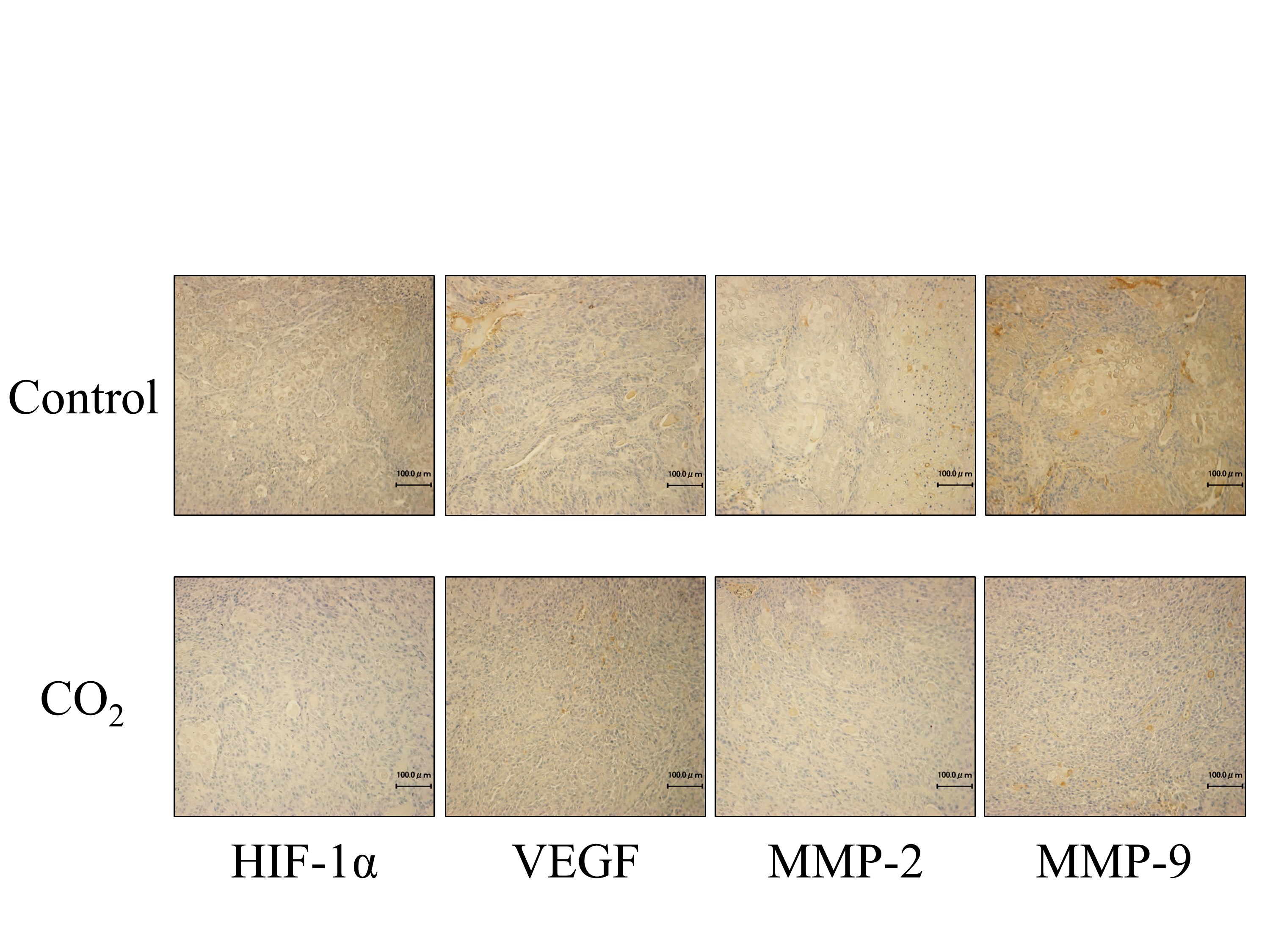

Supplement: Figure S1 — The effect of transcutaneous CO2 on the metastatic potential of HSC-3. In an enlarged image of immunohistochemical staining, we observed that hypoxic condition (HIF-1α), abnormal excessive vascularization (VEGF) and metastatic potential (VEGF, MMP-2 and MMP-9) were decreased in CO2-treated cancer cells. (TIF) [file pone.0100530.s001.tif]
